# Component Profiling of Soy-Sauce-Like Seasoning Produced from Different Raw Materials

**DOI:** 10.3390/metabo10040137

**Published:** 2020-04-01

**Authors:** Tomoyuki Yamana, Moyu Taniguchi, Takeharu Nakahara, Yusuke Ito, Natsuki Okochi, Sastia Prama Putri, Eiichiro Fukusaki

**Affiliations:** 1 Department of Biotechnology, Graduate School of Engineering, Osaka University, 2-1 Yamadaoka, Suita, Osaka 565-0871, Japan; tomoyuki_yamana@bio.eng.osaka-u.ac.jp (T.Y.); moyu_taniguchi@bio.eng.osaka-u.ac.jp (M.T.); sastia_putri@bio.eng.osaka-u.ac.jp (S.P.P.); 2 Research and Development Division, Kikkoman Corporation, 338 Noda, Noda, Chiba 278-0037, Japan; tnakahara@mail.kikkoman.co.jp (T.N.); yusito@mail.kikkoman.co.jp (Y.I.); nmatsumura@mail.kikkoman.co.jp (N.O.)

**Keywords:** soy-sauce-like seasoning, component profiling, umami

## Abstract

Soy sauce is a traditional Japanese umami seasoning commonly made from soybeans, wheat, and salt water. Soy-sauce-like seasoning, made from other raw materials, such as rice and peas, has recently been developed. However, differences in the taste of soy-sauce-like seasoning, depending on the raw materials, have not been evaluated. Component profiling based on GC/MS combined with a paired comparison test were used to investigate the effect of raw materials on seasoning components and umami taste in five grain-based and four bean-based soy-sauce-like seasonings. In a principal component (PC) analysis, grain-based samples and bean-based samples were separated along the PC1 axis (explaining 48.1% of the total variance). Grain-based samples had a higher saccharide content, and bean-based samples had a higher amino acid content. Furthermore, differences in the umami intensity were also observed among sample types. This is the first detailed metabolomics study of the characteristic compounds and umami of a variety of soy-sauce-like seasonings made from different raw materials.

## 1. Introduction

Soy sauce, a traditional Japanese fermented seasoning, is essential for conferring umami taste to Japanese dishes. According to the Japanese Agricultural Standard (JAS), soy sauce is defined as a fermented seasoning made by cooking soybeans and grain (wheat), growing koji mold, and adding salt water [1]. Accordingly, the raw materials in soy sauce are soybeans, grains, and salt water.

To meet consumer demands, soy-sauce-like seasonings [2,3,4] have recently been developed. In these alternative seasonings, soybeans and wheat are replaced with a single type of bean, such as peas or broad beans, or a single type of grain, such as rice or foxtail millet, as raw materials.

The characteristic aromatic compounds and the effect of microbes on soy sauce flavor compounds have been investigated in ordinary Japanese soy sauce made from soybeans and wheat [5,6]. However, few studies have focused on the flavor of soy-sauce-like seasoning. Additionally, nitrogen content has been measured using the Kjeldahl method to evaluate the quality of soy-sauce-like seasoning [7], salt content has been measured using the Mole method [8], and reducing sugar and amino acid analyses [2] have been performed. However, these analysis methods do not clearly reveal differences in flavor among soy-sauce-like seasonings made with different raw materials. Therefore, comprehensive component analyses and sensory evaluations are necessary to investigate the effect of raw materials on the flavor of soy-sauce-like seasonings.

Metabolomics is an omics approach based on the comprehensive analysis of metabolites; it is used in a wide variety of fields, such as medical research, microbiology, and food science [9]. In food research, metabolomics is combined with sensory evaluations to investigate correlations between metabolites and flavor, providing the ability to simultaneously analyze low-molecular-weight compounds related to flavor [10]. In contrast to targeted analyses of specific components, non-targeted or wide-targeted analyses by GC/MS are used to investigate many kinds of components and to understand sample characteristics based on component profiles [11]. Metabolomics approaches have been applied not only to soy sauce but also to cheese [12], coffee [13], and other food products [14,15]. In soy sauce research, correlations between hydrophilic low-molecular-weight compounds and dipeptides and sensory profiles have been investigated [16,17]. These previous studies demonstrate the effectiveness of metabolomics based on GC/MS for investigating the flavor of soy sauce.

In this study, a metabolomics analysis and sensory evaluation were used to evaluate soy-sauce-like seasonings made from only one type of bean or grain as a raw material. This is the first investigation of the effect of raw materials on the component profile and taste of soy-sauce-like seasonings.

## 2. Results and Discussion

### 2.1. Differences in Components between Soy-Sauce-Like Seasonings Made from Different Grains and Beans

**Figure 1:**
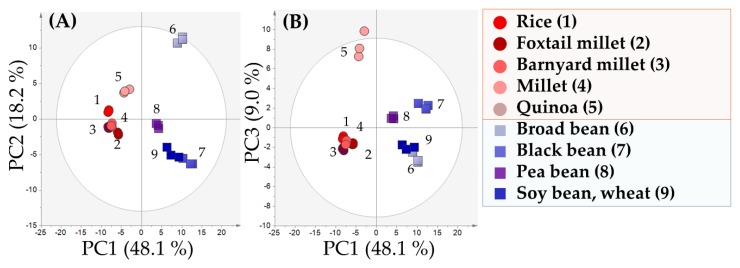
Principal component analysis (PCA) score plot of hydrophilic, low-molecular-weight compounds based on gas chromatography–mass spectrometry (GC/MS). (**A**) PCA score plot (PC1 and PC2). Points and labels indicate samples and sample number, respectively. Circles indicate grain-based samples, and hexagons indicate bean-based samples. (**B**) PCA score plot (PC1 and PC3).

A GC/MS analysis of soy-sauce-like seasoning was conducted to obtain a global view of compounds in samples. In particular, GC/MS-based component profiling of low-molecular-weight, hydrophilic compounds was performed. A total of 133 peaks were annotated in the soy-sauce-like seasonings, including fifteen unknown compounds. Using these 133 compounds as explanatory variables, a principal component analysis (PCA) was conducted, as shown in Figure 1. In the PCA, PC1 explained 48.1% of the total variance, and PC2 and PC3 explained 18.2% and 9.0% of the variance, respectively. A total of 75.3% of the variance among samples was explained by the PCA. In the score plot (Figure 1A), soy-sauce-like seasonings made from beans were located on the positive side, and samples made from grain were located on the negative side. The loading plot was used to investigate characteristic compounds contributing to the sample separation. In the loading plot, each compound was plotted as an explanatory variable. Compounds with higher factor loadings (as absolute values) corresponded to the samples plotted with a higher absolute value in the score plot. Compounds with factor loadings (as absolute values) greater than 0.1 for each PC are shown in Table 1. Soy-sauce-like seasonings made from grain contained high amounts of saccharides, such as glucose and trehalose, and those made from beans contained high amounts of amino acids, such as glycine, valine, and glutamic acid. According to the Standard Tables of Food Composition in Japan (Table 2), grains are rich in carbohydrates, and beans are rich in protein. During the fermentation of soy sauce, carbohydrates are decomposed to saccharides by amylases, and protein is decomposed to amino acids by proteases [18]. The carbohydrate and protein content in raw materials affects the amounts of saccharides and amino acids in soy-sauce-like seasoning. These results suggest that soy-sauce-like seasoning made from raw materials with high carbohydrate content has a high saccharide content, and soy-sauce-like seasoning made from raw materials with high protein content contains more amino acids.

### 2.2. Characteristic Components of Broad-Bean-Based Soy-Sauce-Like Seasoning

According to the PCA score plot in Figure 1A, samples made from broad beans were located on the positive side of PC2. In the loading plot, *meso*-erythritol, mannitol, and galactitol were on the positive side of PC2 (Table 3). Onishi et al. [19] previously reported that broad-bean-based soy-sauce-like seasoning contains some sugar alcohols, such as mannitol and erythritol. In addition, ornithine has been reported as a characteristic compound in broad-bean-based soy-sauce-like seasoning, consistent with our results. A previous study showed that lactic acid bacteria can break down arginine during fermentation [20]. This suggests that ornithine was produced by lactic acid bacteria, which was able to break down arginine in broad-bean-based soy-sauce-like seasoning.

### 2.3. Characteristic Components of Quinoa-Based Soy-Sauce-Like Seasoning

In the PCA score plot shown in Figure 1B, there was a clear separation between soy-sauce-like seasoning made from quinoa, on the positive side of PC3, and other samples. Moreover, in the loading plot (Table 4), tryptophan and ribose were plotted on the positive side of PC3. According to a previous study of quinoa [21], ratios of d-ribose in carbohydrates and tryptophan in proteins are high. Therefore, the detected compounds were considered characteristic components of quinoa-based soy-sauce-like seasoning because of the decomposition of carbohydrates and proteins by amylases and proteases. Based on these results, characteristics of raw materials influence the components of soy-sauce-like seasoning. In addition, PC4 and PC5 contributed to the separation of black beans, pea beans, and soybeans (Figure S1).

### 2.4. Differences in Umami and Sweetness Depending on Raw Materials Based on Paired Comparisons 

**Figure 2:**
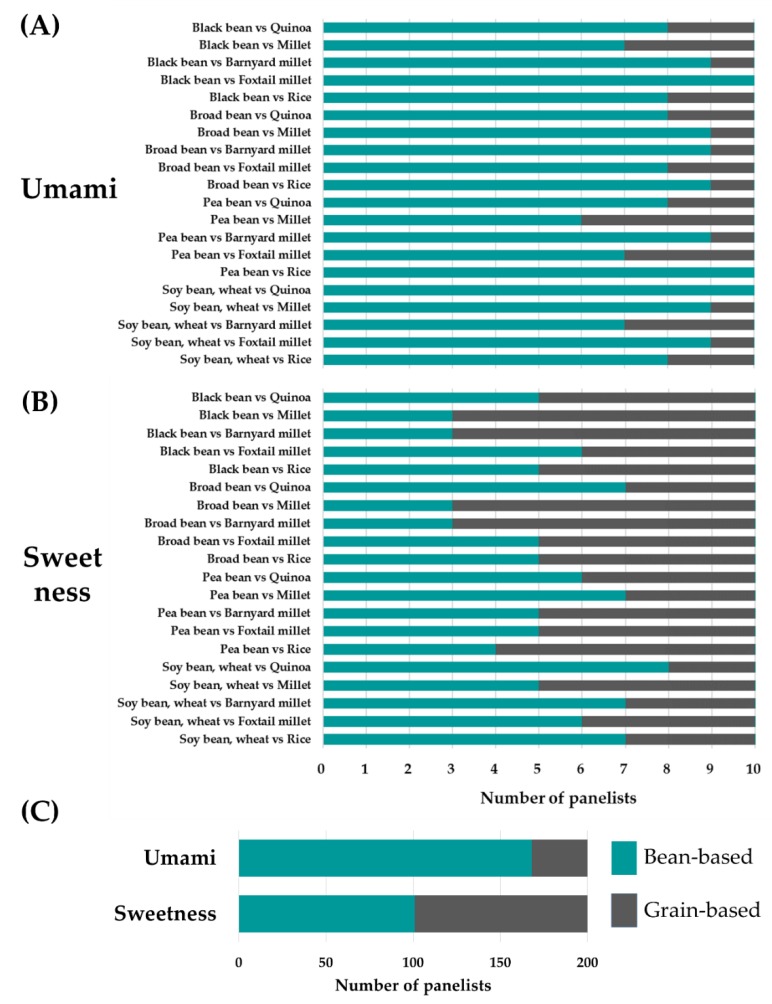
Paired comparisons of the intensities of umami and sweetness between grain-based and bean-based samples. (**A**), (**B**) Number of panelists that selected each sample in pairwise comparisons of soy-sauce-like seasonings made from grain and beans. (**C**) Number of panelists that selected soy-sauce-like seasonings made from grain and beans based on umami and sweetness.

In the PCA, PC1 explained 48.1% of the total variance among samples. This indicates that the components of PC1 contribute to the differences among soy-sauce-like seasonings made from different raw materials. In addition, the loading plot showed that samples made from grain contained large quantities of saccharides, and samples made from beans contained abundant amino acids. To investigate whether the differences in component profiles influence taste, paired comparisons were performed using samples made from grains and beans. The analysis focused on umami and sweetness for two reasons. First, enhancing the umami taste of foods is an important role of soy sauce. Second, based on the PCA, saccharides associated with sweetness were characteristic components of grain-based samples, and various amino acids associated with umami were characteristic components of bean-based samples. The results of the paired comparisons are summarized in Figure 2. Figure 2A,B shows the poll results that explain how many panelists selected bean samples or grain samples in each pair. As shown in Figure 2A, bean-based samples tended to have a more intense umami taste than grain-based samples. Furthermore, Figure 2C shows the total poll results, which explain how many panelists selected the bean group or the grain group in a total of 20 pairs based on umami and sweetness. In total, 84% of the panelists indicated that bean-based samples had a stronger umami taste, with a significant difference between grain-based and bean-based samples (binomial test, *p* < 0.01). Bean-based samples are predicted to have a stronger umami taste than grain-based samples because of the presence of glutamic acid [15], which was detected as a characteristic component of bean-based samples in the PCA. However, the panelists detected no significant difference in sweetness between sample types (Figure 2B,C).

In conclusion, to investigate the effects of raw materials on the components and taste of soy-sauce-like seasonings, hydrophilic, low-molecular-weight compounds were evaluated by a metabolomics approach, and paired comparisons of grain-based and bean-based samples with respect to umami and sweetness were performed. PCA results showed that there was a clear separation between soy-sauce-like seasonings made from beans and grain. Within these groups, grain-based samples were characterized by saccharides, such as glucose and trehalose, while bean-based samples were characterized by amino acids, such as glutamic acid. Additionally, for soy-sauce-like seasonings made from broad beans and quinoa, the characteristics of the raw material are expected to influence the characteristic components of soy-sauce-like seasoning. Sensory evaluation by means of paired comparison showed that bean-based samples had a stronger umami taste than grain-based samples. Accordingly, it is possible that the carbohydrate and protein content in raw materials determine the component profiles of soy-sauce-like seasoning and have an effect on the umami intensity. This is the first detailed study of the characteristic components and taste of a variety of soy-sauce-like seasonings made from different raw materials. The results of this study give the industry new insights to help in the selection of raw materials for developing a new soy sauce that would meet various consumer needs. In addition, this study can be used as a reference to expand the taste and variety of soy sauce by using different raw materials.

## 3. Materials and Methods 

### 3.1. Soy-Sauce-Like Seasoning Sample

Soy-sauce-like seasonings made using only bean or grain and salt were purchased as samples (Table 5). All samples were stored at 4 °C.

### 3.2. Reagents

Ribitol and pure pyridine were purchased from Wako Pure Chemical Industries (Osaka, Japan). Methoxyamine hydrochloride was purchased from Sigma-Aldrich Japan (Tokyo, Japan). *N*-Methyl-*N*-(trimethylsilyl), trifluoroacetamide (MSTFA), and alkane mix (C9–C40) were purchased from GL Science (Tokyo, Japan).

### 3.3. Derivatization of Hydrophilic, Low-Molecular-Weight Compounds for GC/MS Analysis

Soy-sauce-like seasoning samples were diluted 10-fold with ultrapure water. Next, 20 µL of diluted sample was dispensed into a 1.5 mL microfuge tube. Then, 60 µL of ribitol (0.2 mg/mL in ultrapure water) was added to the sample as an internal standard. Then, the mixture was lyophilized. Following that, 100 mL of methoxyamine hydrochloride in pyridine (20 mg/mL) was added for derivatization after a freeze-dry process. To induce the methoxylation reaction, the mixture was incubated in a shaker incubator (Eppendorf Ltd., Hamburg, Germany) at 30 °C for 90 min. For silylation reaction, 50 µL of MSTFA were added, and the mixture was incubated at 37 °C for 30 min. Finally, the derivatized solutions were transferred to vials for GC/MS analysis. In this experiment, each soy-sauce-like seasoning sample was analyzed in triplicate for technical replicate (*n* = 3).

### 3.4. GC/MS Analysis for Hydrophilic Compounds

In this study, GCMS-QP2010 Ultra (Shimadzu, Kyoto, Japan), equipped with a 30 m × 0.25 mm i.d. fused silica capillary column coated with 0.25 mm InertCap 5MS/NP (GL science, Tokyo, Japan), was used for analysis. The analytical conditions for hydrophilic compound analysis used were as follows: 1 µL of the derivatized sample was injected by 1 µL to GC/MS with split mode (25:1 (*v/v*)). The injection temperature was 230 °C. The carrier gas was helium at a linear velocity of 39 cm/s. The column temperature was held at 80 °C for 2 min isothermally, and then raised at 15 °C/min to 330 °C and held for 6 min. The transfer line and the ion source temperatures were 250 and 200 °C, respectively. Ions were generated by electron ionization (EI) at 70 eV. Mass spectra were recorded at 6.67 scans per second over the mass range of *m/z* 85–500. Order of the sample analysis was set randomly by using Excel’s RAND function. A standard alkane mixture (C9−C40) was injected at the beginning of the analysis to calculate retention indices (RIs) used for tentative identification.

### 3.5. Data Analysis

The obtained raw data from GC/MS analysis were converted to the netCDF format by using GC/MS solution software (Shimadzu, Kyoto, Japan). Baseline correction, noise removal, peak detection, and alignment were done using a freely available software MetAlign [22]. Afterwards, data matrix was constructed based on processed data exported from MetAlign using AIoutput2 annotation software [23]. By comparing the retention indices (RIs) and their EI mass spectra with our in-house library prepared using authentic standards, the tentative peak identification was performed with AIoutput2. RIs of each peak were calculated based on retention time of standard n-alkane mixture. To confirm the results of annotations, NIST11 MS spectral library was used. The assigned peak intensities were normalized against the peak intensities of ribitol, which was added as an internal standard.

### 3.6. Investigation of Difference on Umami, Sweetness among Different Raw Material Based on Paired Comparison

The difference in umami and sweetness between bean-based and grain-based soy-sauce-like seasoning samples was investigated using a paired comparison test. Paired comparison is one of the most popular sensory tests. By using this method, panelists evaluate only two samples at a time, which significantly reduces the effect of fatigue, carryover, and memory compared to evaluating more than two samples at the same time. In this experiment, the same nine samples with GC/MS analysis were used for the paired comparison test. Each pair consisted of one bean-based sample (four types) and one grain-based sample (five types). In total, 20 pairs were tested in this experiment. Because the color of each sample was a little different, color was considered when determining what might influence and bias the panelists. For this reason, the color of each sample was hidden by using black plastic cups and black tea spoons. Ten milliliters of each sample was served to each panelist, and the samples were evaluated by 20 semitrained panelists. The semitrained panelists, who were between the ages of 22 to 27, were trained using the five basic taste test. Each pair was evaluated by 10 panelists, who were selected randomly from 20 panelists. The panelists then selected which sample tasted stronger based on umami and sweetness. Before and after sample tasting, panelists used mineral water to rinse their mouths to prevent carry-over of taste.

## Figures and Tables

**Table 1:** Characteristic hydrophilic low-molecular-weight compounds in grain-based and bean-based soy-sauce-like seasonings contributing to PC1.

**(A)Compound**	***p(corr)*[PC1]**	**Compound**	***p(corr)*[PC1]**
β-Alanine	0.1242	Galactose	0.1174
Phenylalanine	0.1241	Allose	0.1174
β-*N*-Methyl amino alanine	0.1237	Hypoxanthine	0.1168
Glycine	0.1237	Dihydroxyacetone	0.1160
Isoleucine	0.1237	Unknown	0.1142
Valine	0.1235	Inositol	0.1136
Threonine	0.1234	Xylonic acid	0.1116
Lysine	0.1234	Alanine	0.1107
Phosphoric acid	0.1232	Pyroglutamic acid	0.1100
Leucine	0.1230	Tyrosine	0.1097
Maleic acid	0.1228	Xanthine	0.1084
Glutamic acid	0.1227	Histidine	0.1077
5-Aminovaleric acid	0.1225	Cysteine	0.1050
*N*-α-Acetyl ornithine	0.1223	Unknown	0.1049
*N*-α-Acetyl lysine	0.1215	Xylitol	0.1047
Proline	0.1215	Alanylalanine	0.1045
Normetanephrine	0.1211	Methionine	0.1040
2-Aminoethanol	0.1203	Unknown	0.1039
2-Aminoadipic acid	0.1203	Uracil	0.1033
Glycolic acid	0.1197	Malonic acid	0.1022
Serine	0.1197	Mannose	0.1021
Thymine	0.1186	Phthalic acid	0.1007
Lyxose	0.1184	-	-
**(B)Compound**	***p(corr)*[PC1]**	**Compound**	***p(corr)*[PC1]**
Glucose	−0.1193	*N*-Acetyl galactosamine	-0.1091
Unknown	−0.1161	Trehalose	-0.1067
Glyceryl-glycoside	−0.1148	β-Lactosebe	-0.1038
Thymidine	−0.1131	Maltose	-0.1036
Melibiose	−0.1107	Lactitol	-0.1034

(**A**) Compounds plotted on the positive side (bean-based side) along PC1 and factor loadings are shown. (**B**) Compounds plotted on the negative side (grain-based side) along PC1 and factor loadings are shown. Compounds with factor loadings of greater than ±0.1 were selected as standards.

**Table 2:** Protein and carbohydrate content in raw materials (from the Japanese Standard Tables of Food Composition in Japan 2015).

Raw Material Name	Protein (g) *a	Carbohydrate (g) *b
Grain/Rice/Milled grain	6.1	77.6
Grain/Foxtail millet/Milled grain	11.2	69.7
Grain/Bamyard millet/Milled grain	9.4	73.2
Grain/Millet/Milled grain	11.3	70.9
Grain/Quinoa/Brown grain	13.4	69.0
Grain/Wheat/Brown grain	10.6	72.2
Bean/Broad bean/Dry	26.0	55.9
Bean/Pea bean/Dry	21.7	60.4
Bean/Black bean/Dry	33.9	30.8
Bean/Soy bean/Dry	33.8	29.5

*a: Calculated as the product of the quantitative nitrogen content measured by the improved Kjeldahl method and nitrogen–protein conversion factor. *b: Calculated by the subtraction method (i.e., total amounts of moisture, protein, lipids, and ash were subtracted from 100 g).

**Table 3:** Characteristic hydrophilic low-molecular-weight compounds in broad-bean-based soy-sauce-like seasoning on PC2.

Compound	*p(corr)*[PC2]	Compound	*p(corr)*[PC2]
Urea	0.1743	Myristic acid	0.1563
Putrescine	0.1742	2-Hydroxybutyric acid	0.1437
4-Aminobutyric acid	0.1735	Lactic acid	0.1409
Butanoic acid	0.1735	Galactitol	0.1375
Ornithine	0.1708	Arabitol	0.1301
Histamine	0.1693	Galacturonic acid	0.1274
Mannitol	0.1693	Sorbitol	0.1154
Cystamine	0.1665	Guanine	0.1138
*meso*-erythritol	0.1663	Oxaloacetic acid+Pyruvic acid	0.1056
Glycylglycine	0.1627	Oxalic acid	0.1008
Tyramine	0.1577	-	-

Compounds on the positive side (broad-bean side) of PC2 and factor loadings are shown. Compounds were selected based on factor loadings of over 0.1 as standards.

**Table 4:** Characteristic hydrophilic low-molecular-weight compounds in quinoa-based soy-sauce-like seasoning on PC3.

Compound	*p(corr)*[PC3]	Compound	*p(corr)*[PC3]
Unknown	0.2645	Xylulose+Ribulose	0.1989
Cadaverine	0.2603	Acetoacetic acid	0.1487
2-Aminobutyric acid	0.2578	Tryptophan	0.1282
Psicose+Tagatose	0.2574	Lauric acid	0.1153
3-Hydroxybutyric acid	0.2512	Unknown	0.1143
Ribose	0.2512	2-Hydroxybutyric acid	0.1109
Galacturonic acid	0.2037	Xanthine	0.1092
Oxalic acid	0.2003	Gluconic acid	0.1011

Compounds on the positive side (i.e., the side including quinoa) of PC3 and factor loadings are shown. Compounds were selected based on factor loadings of over 0.1 as standards.

**Table 5:** Soy-sauce-like seasoning sample list.

Sample Number	Sample Name	Raw Materials	Classification
1	Rice *shoyu*	Rice	Grain
2	Awa *shoyu*	Foxtail millet	
3	Hie *shoyu*	Barnyard millet	
4	Kibi *shoyu*	Millet	
5	Quinoa *shoyu*	Quinoa	
6	Soramame *shoyu*	Broad bean	Bean
7	Kuromame *shoyu*	Black bean	
8	Endomame *shoyu*	Pea bean	
9	Marudaizu *shoyu*	Soy bean, wheat	

Sample name, raw materials, and classification of raw materials of soy-sauce-like seasoning made from a single type of bean or grain are indicated.

## Supplementary Materials

The following are available online at https://www.mdpi.com/2218-1989/10/4/137/s1, Figure S1: PCA score plot using different principal components from PC1 ~ PC5.
