# Supplementary figures and images for: Component Profiling of Soy-Sauce-Like Seasoning Produced from Different Raw Materials
Source: Metabolites. 2020 Apr 1;10(4):137. doi: 10.3390/metabo10040137 (PMC7240962; doi:10.3390/metabo10040137)

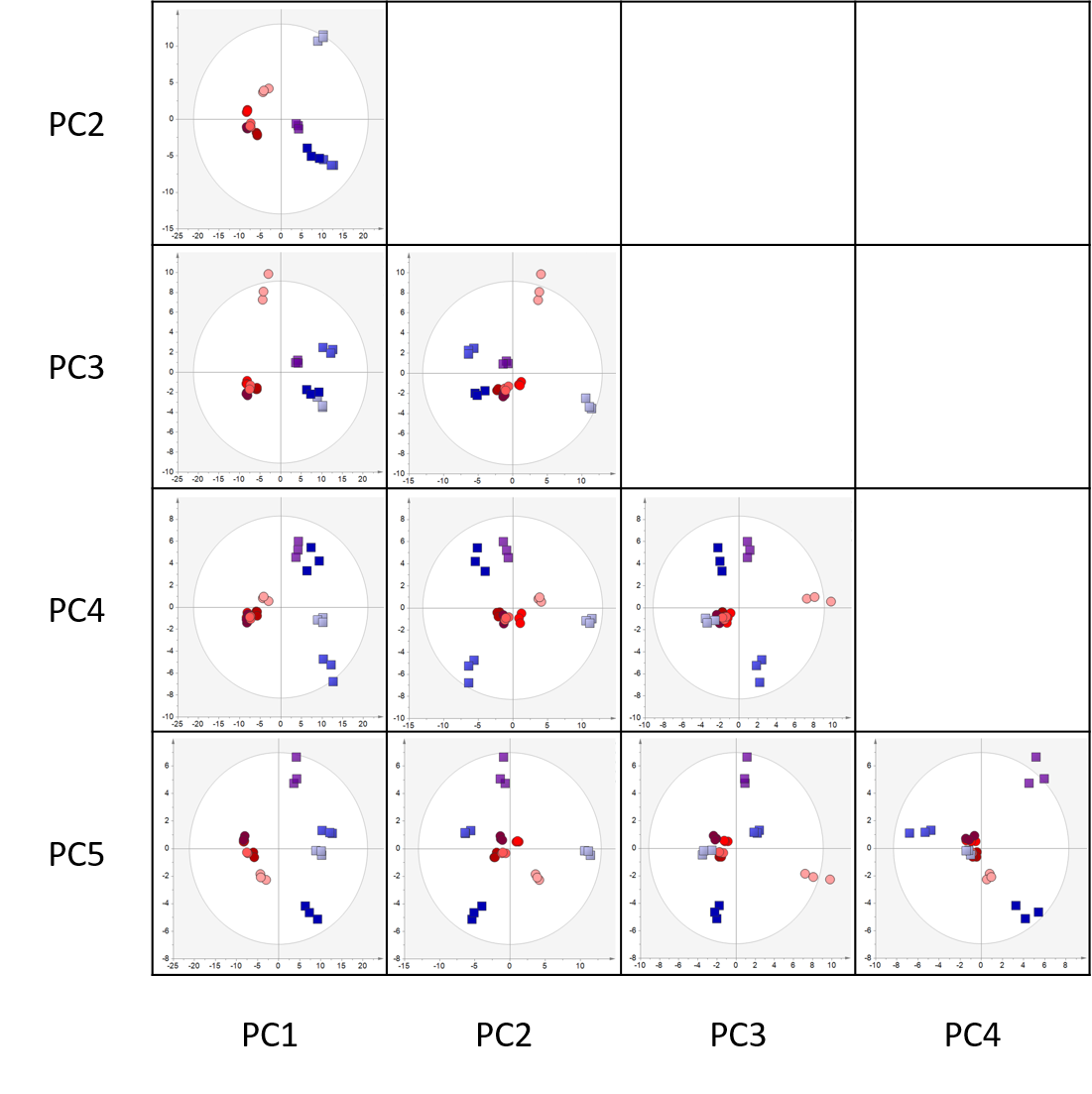

Supplement: Supplementary file 1 [file metabolites-10-00137-s001.zip › FigureS1.png]
